# Supplementary figures and images for: Loss of neuropeptidergic regulation of cholinergic transmission induces homeostatic compensation in muscle cells to preserve synaptic strength
Source: PLoS Biol. 2025 May 8;23(5):e3003171. doi: 10.1371/journal.pbio.3003171 (PMC12088594; doi:10.1371/journal.pbio.3003171)

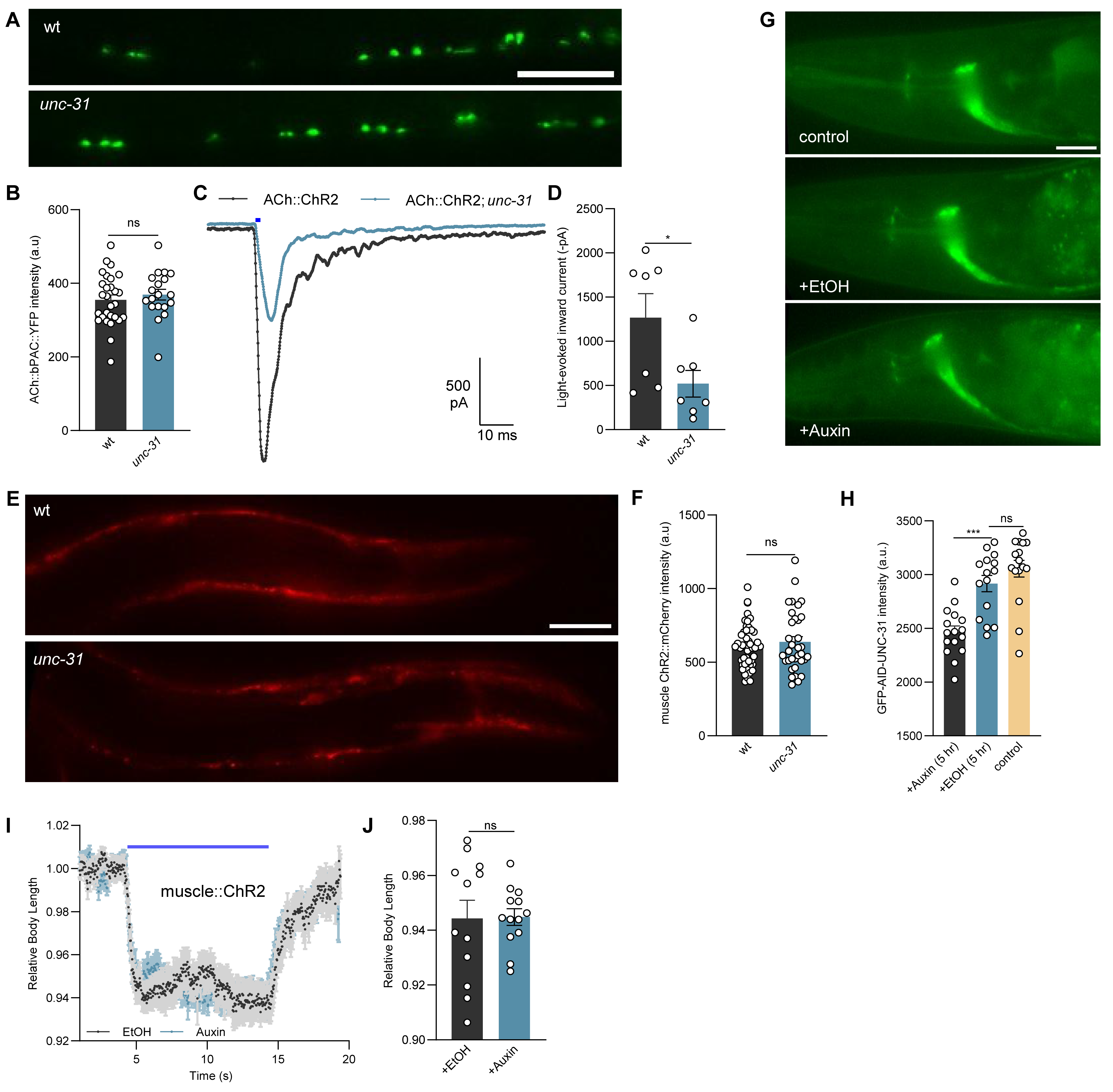

Supplement: S1 Fig — (A, B) Quantification of bPAC::YFP signal in ventral nerve cord cholinergic neurons of wt (n = 28) and unc-31 mutants (n = 19). Representative images (A; Scale bar 10 µm) and summary data (B) are shown. (C, D) Cholinergic ChR2 activation-evoked postsynaptic currents in wt (n = 7) and unc-31 mutants (n = 7). Representative traces (C) and group data of peak currents (D) are shown. (E, F) Quantification of ChR2::mCherry signal in body wall muscles of wt (n = 48) and unc-31 mutants (n = 34). Representative images (E; Scale bar 10 µm) and summary data (F) are shown. (G, H) Quantification of GFP-AID-UNC-31 intensity in nerve ring after ethanol (n = 15) or auxin (n = 15) 5 h exposure compared to control animals (n = 16, no treatment). Representative images (G; Scale bar 10 µm) and summary data (H) are shown. (I, J) Measurements of body length induced by muscular ChR2 activation after UNC-31 depletion using 0.1 mW/mm2 blue light stimulation. Auxin or ethanol treated animals were compared. Animal number tested in each group: n = 12, 13 respectively. Blue bar indicates the 5–15 s blue light illumination. The data in panel J represent the mean values over the entire illumination period shown in panel I. Data presented as mean ± SEM. Statistical significance for two-group datasets and multiple-group datasets comparison was determined using unpaired t test and one-way ANOVA with Tukey-correction respectively. ** and **** indicate p < 0.01 and p < 0.0001, respectively. Numerical data can be found in S1 Dataset. (TIF) [file pbio.3003171.s001.tif]

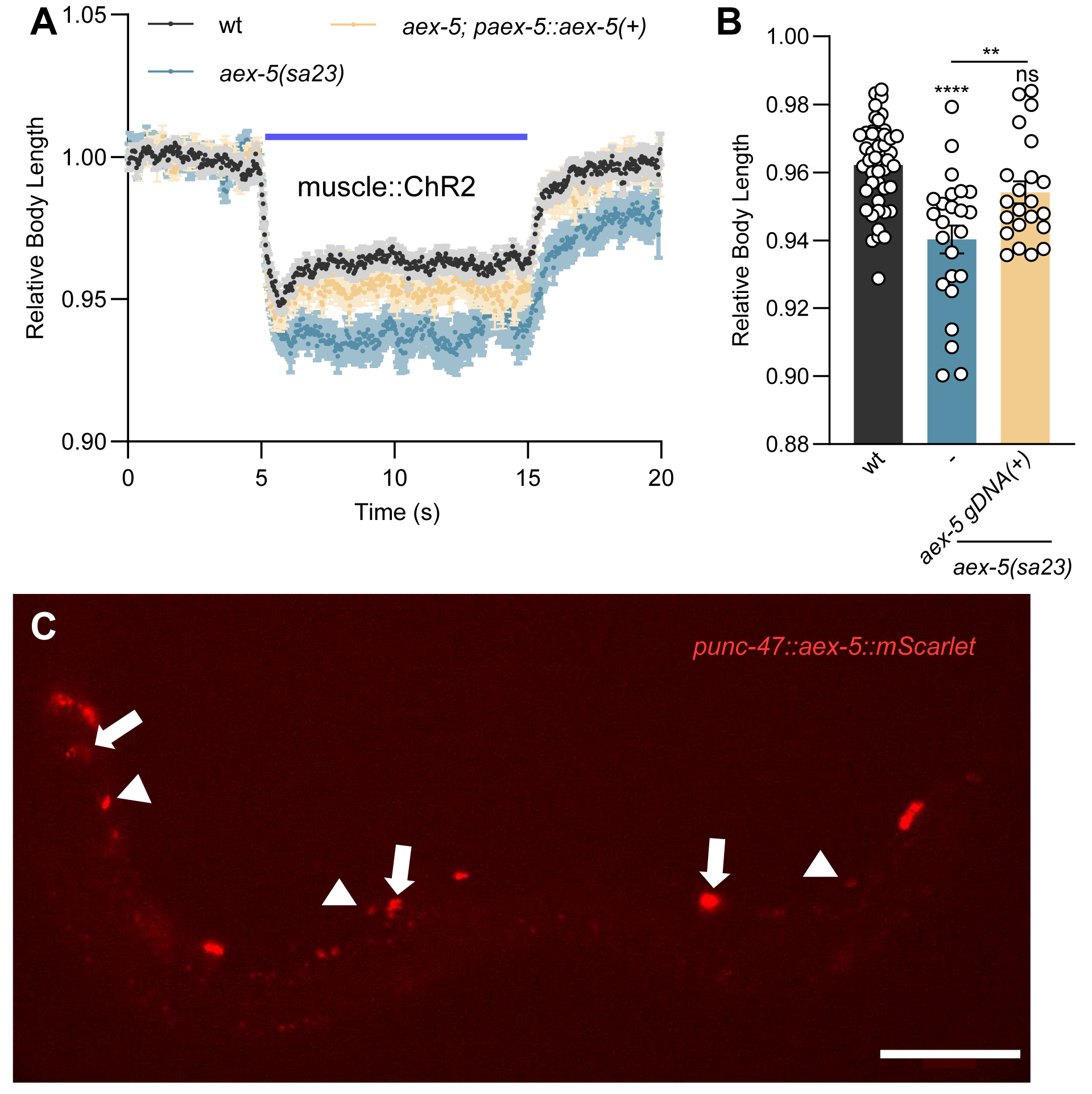

Supplement: S2 Fig — (A, B) Measurements of body length change, induced by muscular ChR2 activation using 65 µW/mm2 blue light stimulation. aex-5 genomic rescue was done by specifically expressing AEX-5 under its own promoter. Animal number tested n = 46, 24, 22, from left to right, respectively. The data in panel B represent the mean values over the entire illumination period (5–15 s) shown in panel A. (C) AEX-5 is released from GABAergic neurons. Coelomocyte fluorescence, resulting from expression in, using the unc-47 promoter, and secretion of mCherry-tagged AEX-5 from GABAergic neurons. White arrows indicate the coelomocytes in the middle and anterior parts of the animal. White arrowheads indicate the ventral cord GABAergic neurons. Scale bar 100 µm. Data presented as mean ± SEM. Statistical significance was determined using one-way ANOVA with Tukey-correction. ** and **** indicate p < 0.01 and p < 0.0001, respectively. Numerical data can be found in S1 Dataset. (TIF) [file pbio.3003171.s002.tif]

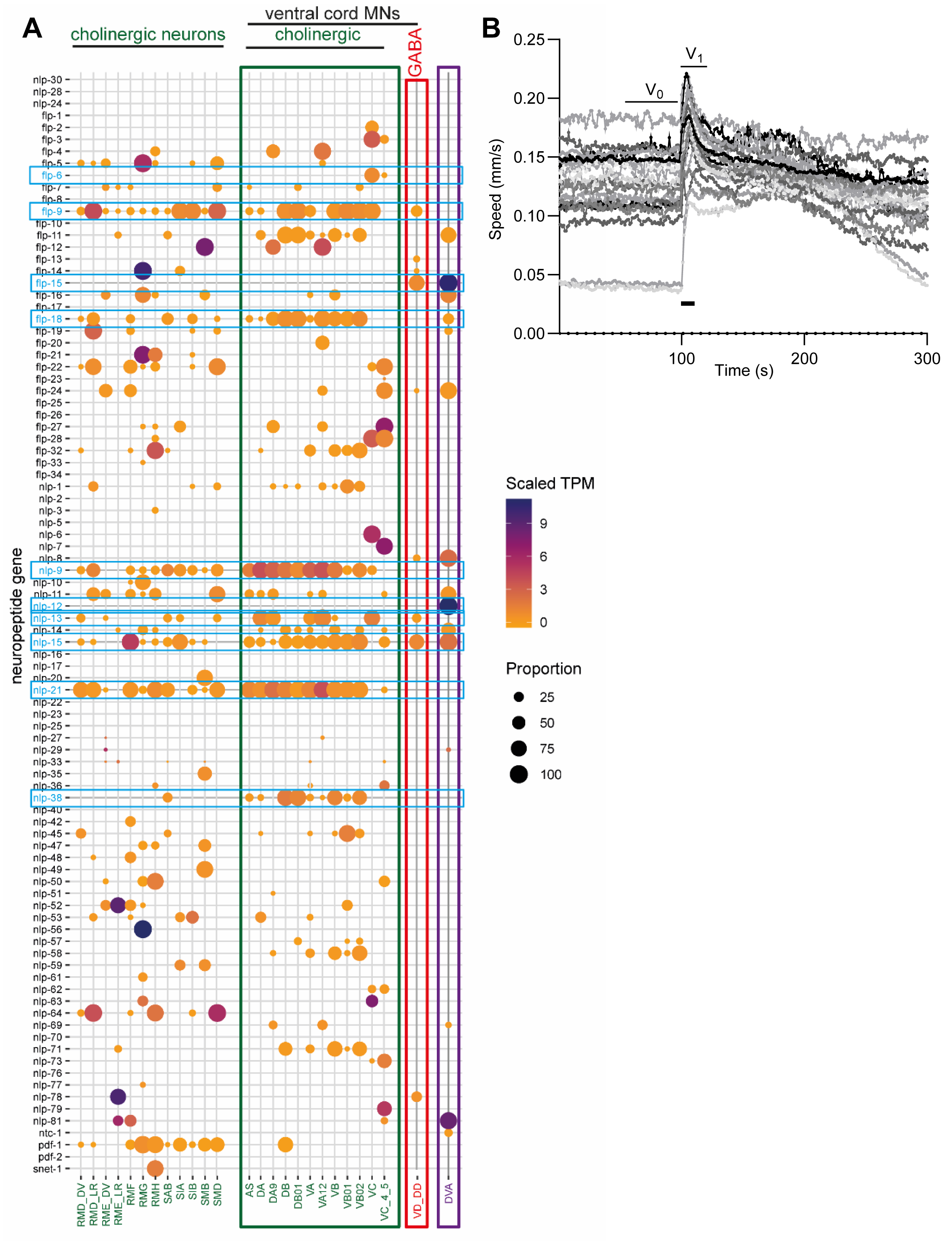

Supplement: S3 Fig — (A) Expression data summary of neuropeptides expressed in MNs. Heatmap representing the expression patterns and levels of neuropeptide mRNAs in different neuron types were generated from CeNGENApp (https://cengen.shinyapps.io/CengenApp/). The initial exported plot was cropped to show cholinergic neurons, GABAergic neurons, and the cholinergic interneuron DVA. Scaled TPM (transcripts per million) and proportion data refer to all neurons of Caenorhabditis elegans, not only the subset shown. (B) Mean speed traces following bPAC stimulation in cholinergic neurons, of the animals indicated in, and giving rise to group data of Fig 3C and 3D. (TIF) [file pbio.3003171.s003.tif]

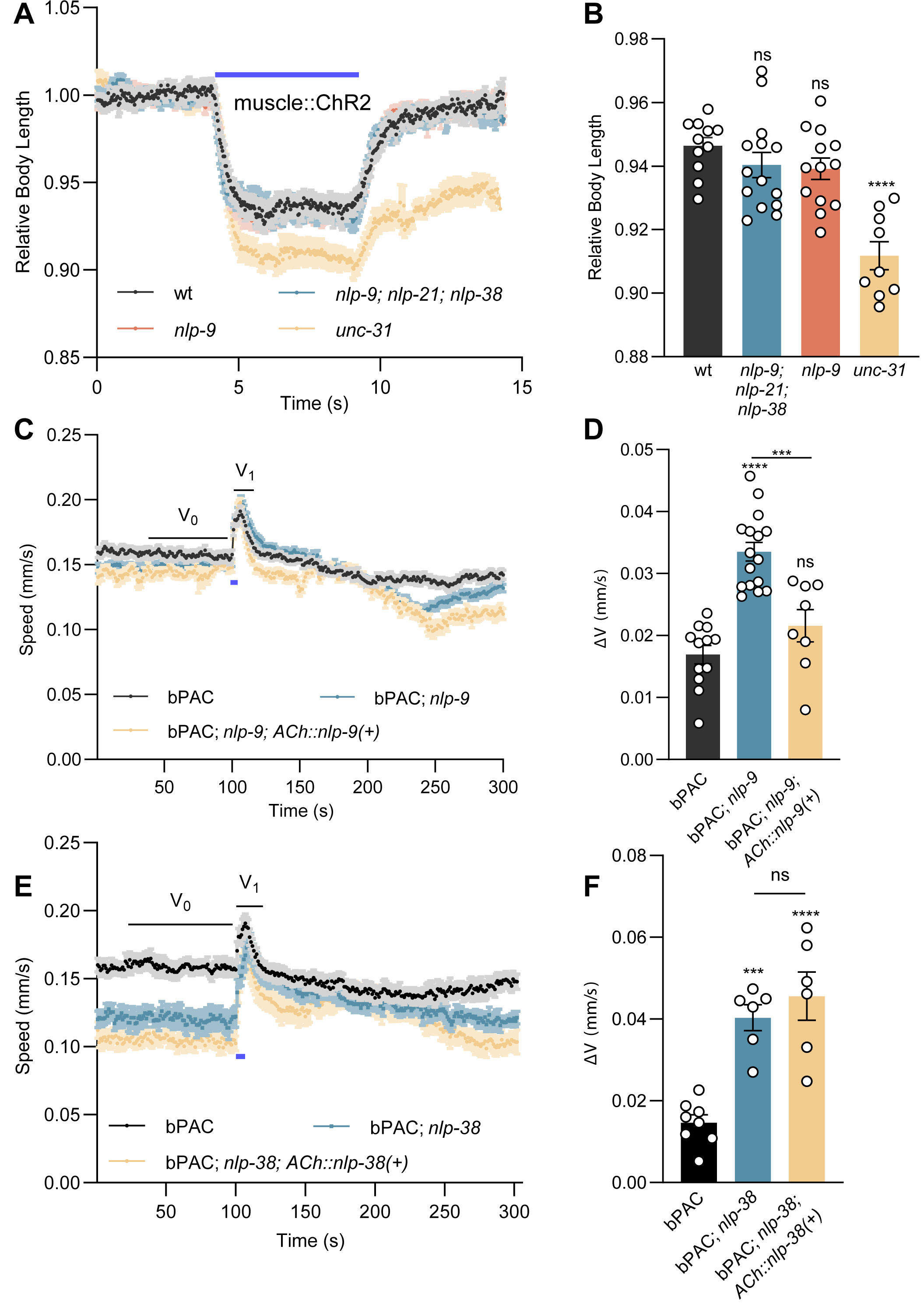

Supplement: S4 Fig — (A, B) Measurements of body length induced by muscular ChR2 activation using 100 µW/mm2 blue light stimulation in wt (n = 11), nlp-9; nlp-21; nlp-38 (n = 14), nlp-9 (n = 13), and unc-31 mutants (n = 9). The data in panel B represent the mean values over the entire illumination period (5–10 s) shown in panel A. (C–F) Crawling speed induced by cholinergic bPAC activation was compared in the indicated genotypes. nlp-9 (C, D) and nlp-38 rescue (E, F) were done by specifically expressing NLP-9 and NLP-38 from the cholinergic promoter unc-17. Animal number tested in (D) n = 60–80, in N = 12, 16, 8 experiments, and in (F) n = 60–80, in N = 8, 6, 6 experiments, from left to right, respectively. All data are presented as mean ± SEM. Statistical significance for two-group datasets and multiple-group datasets comparison was determined using unpaired t test and one-way ANOVA with Tukey-correction respectively. *** and **** indicate p < 0.001 and p < 0.0001, respectively. Numerical data can be found in S1 Dataset. (TIF) [file pbio.3003171.s004.tif]

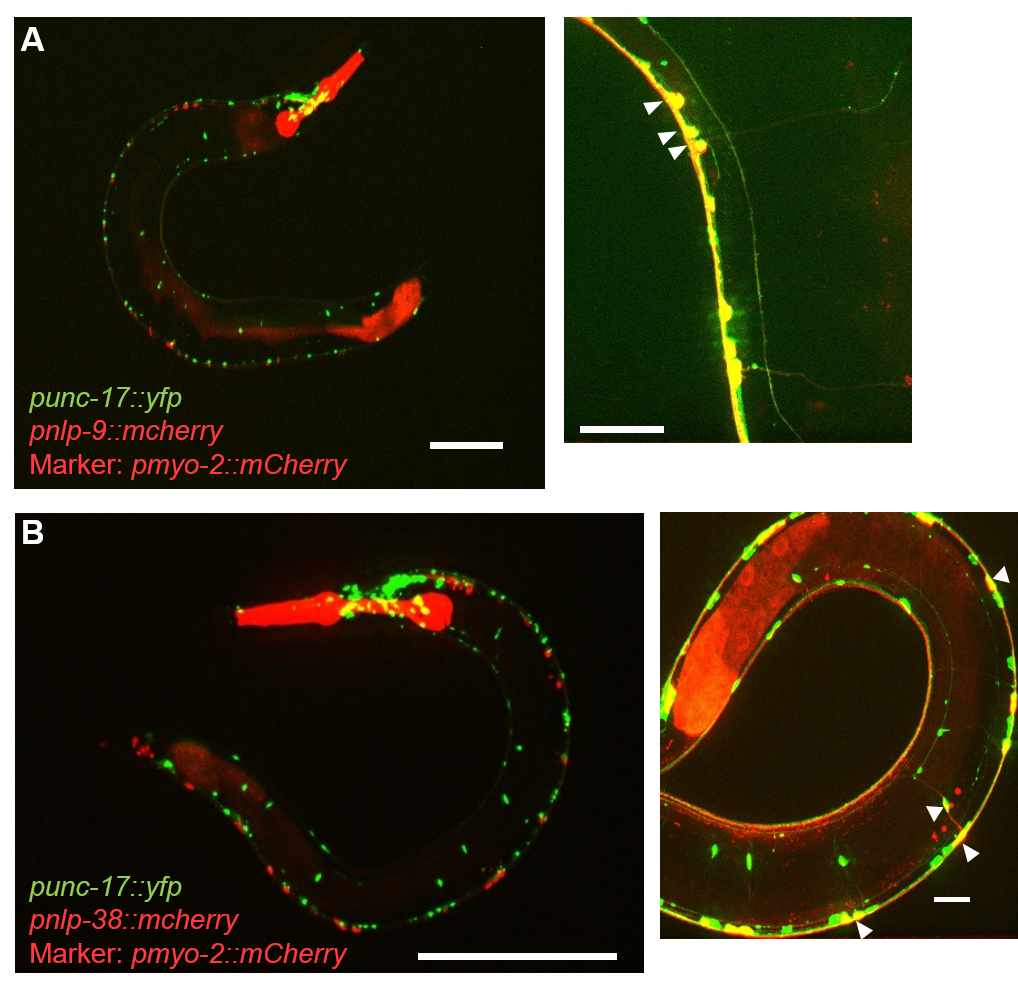

Supplement: S5 Fig — (A, B) Anatomical expression patterns of nlp-9 and nlp-38. Representative images of nlp-9 and nlp-38 transcriptional reporters driving mCherry fluorescence from the nlp-9 and nlp-38 promoters, respectively and their colocalization with the fluorescence of YFP, expressed in cholinergic neurons using the unc-17 promoter. White arrowheads indicate colocalization of cell bodies of nlp-9 or nlp-38 expressing neurons and ventral cord cholinergic MNs. Scale bars 100 and 20 µm, respectively. (TIF) [file pbio.3003171.s005.tif]

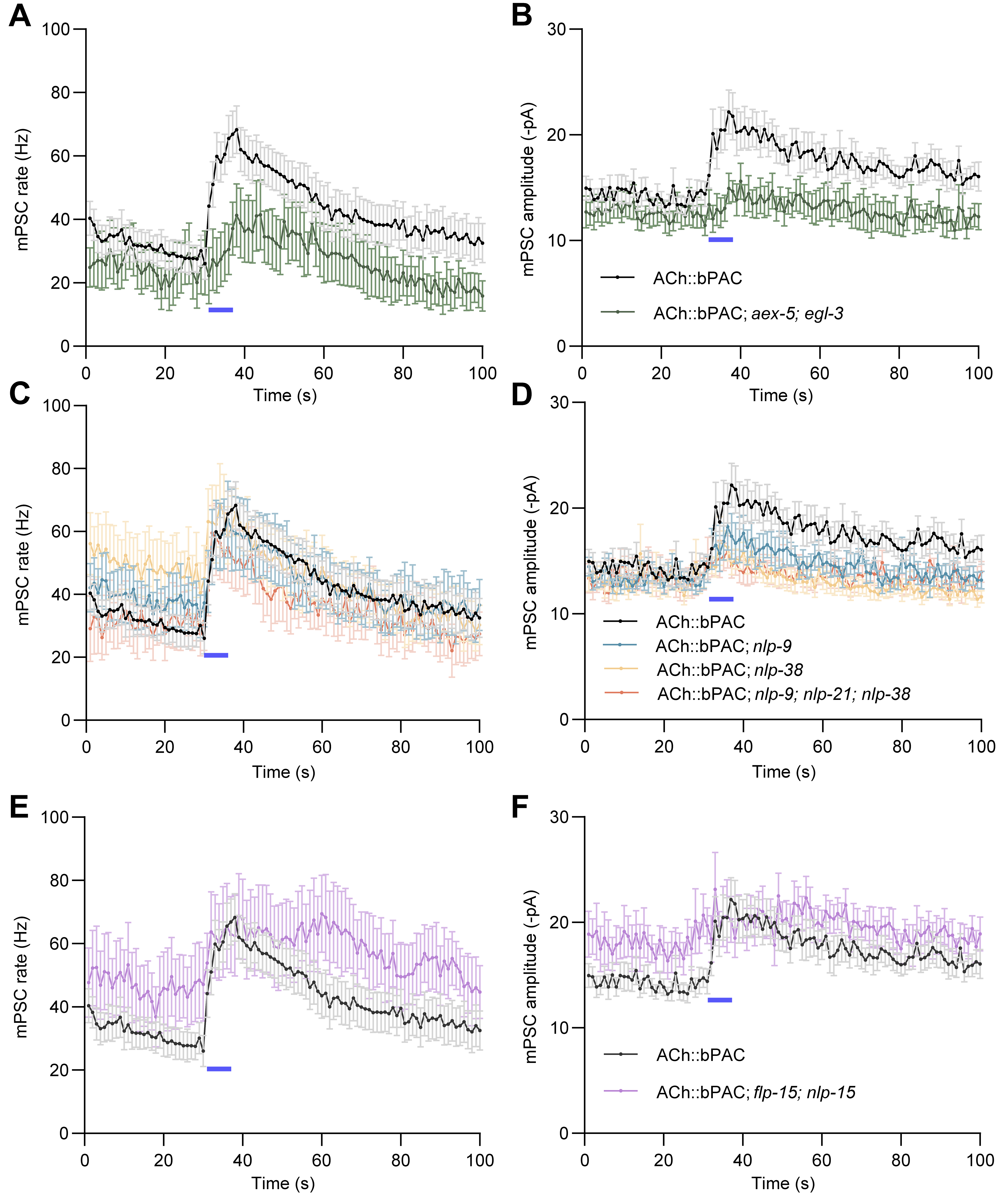

Supplement: S6 Fig — (A–F) Data as in Fig 6B–6G, but not normalized. Analysis of (m)PSC rate and amplitudes in body wall muscle cells, induced by bPAC stimulation of cholinergic neurons. Mean ± SEM mPSCs, recorded from dissected body wall muscle of adult worms in wt (n = 19), aex-5; egl-3 (n = 7), nlp-9 (n = 13), nlp-38 (n = 8), nlp-9; nlp-21; nlp-38 (n = 7), and flp-15; nlp-15 (n = 7) mutants. Blue bar indicates stimulation of bPAC in cholinergic neurons. Note, the same wt control data was used in panels A, C, E and B, D, F. (TIF) [file pbio.3003171.s006.tif]

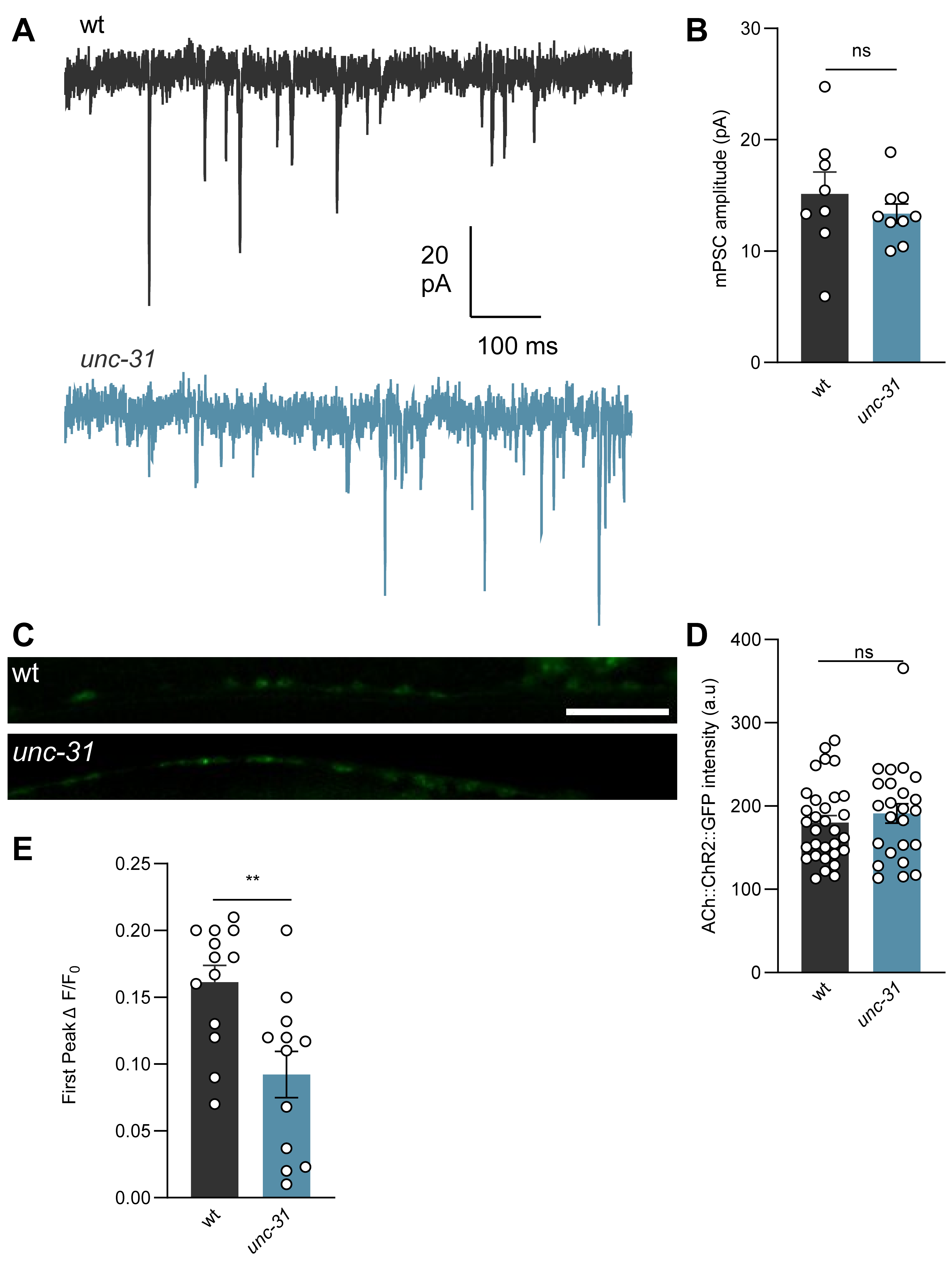

Supplement: S7 Fig — (A, B) Basal, unstimulated mPSCs recorded from dissected BWM cells of adult worms in wt (n = 8) and unc-31 mutants (n = 9). Representative traces of mPSCs (A) and summary data of mPSC amplitudes (B) are shown. (C, D) Quantification of cholinergic ChR2::GFP intensity in ventral nerve cord of wt (n = 31) and unc-31 (n = 24) mutants. Representative images (C; Scale bar 10 µm) and summary data (D) are shown. (E) Voltage imaging using the fluorescent voltage indicator QuasAr, expressed in BWM cells of wt (n = 13) and unc-31 mutants (n = 12), during depolarization evoked by ChR2 stimulation of cholinergic neurons, as in Fig 7G and 7H. The first peak ΔF/F0 is the initial maximal signal observed during ChR2 stimulation. All data are presented as mean ± SEM. Statistical significance comparison was determined using unpaired t test. ns = not significant. Numerical data can be found in S1 Dataset. (TIF) [file pbio.3003171.s007.tif]

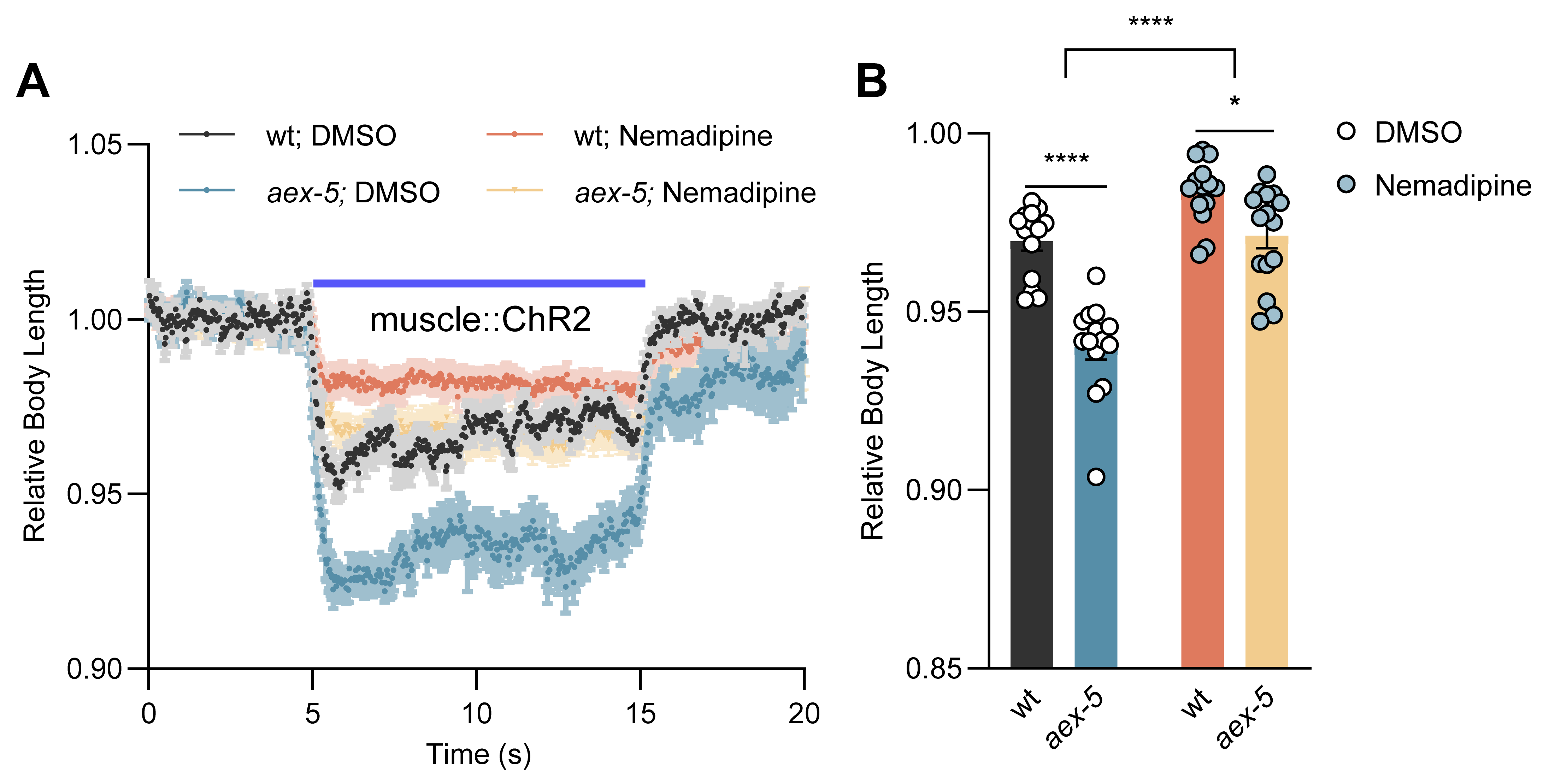

Supplement: S8 Fig — (A, B) Increased evoked muscle contraction in aex-5 mutant is reverted by CaV1 block. Body contraction induced by muscular ChR2 activation using 65 µW/mm2 blue light stimulation was compared in the indicated genotypes and treatments. Relative body lengths after treatment with the CaV1 specific inhibitor nemadipine are shown. Number of animals tested, n = 14, 14, 14, 15, from left to right columns, respectively. The data in panel B represent the mean values over the entire illumination period (5–15 s) shown in panel A. Data are presented as mean ± SEM. Statistical significance comparison was determined using two-way ANOVA with Sidak’s multiple comparisons test. * and **** indicate p < 0.05 and p < 0.0001, respectively. Numerical data can be found in S1 Dataset. (TIF) [file pbio.3003171.s008.tif]

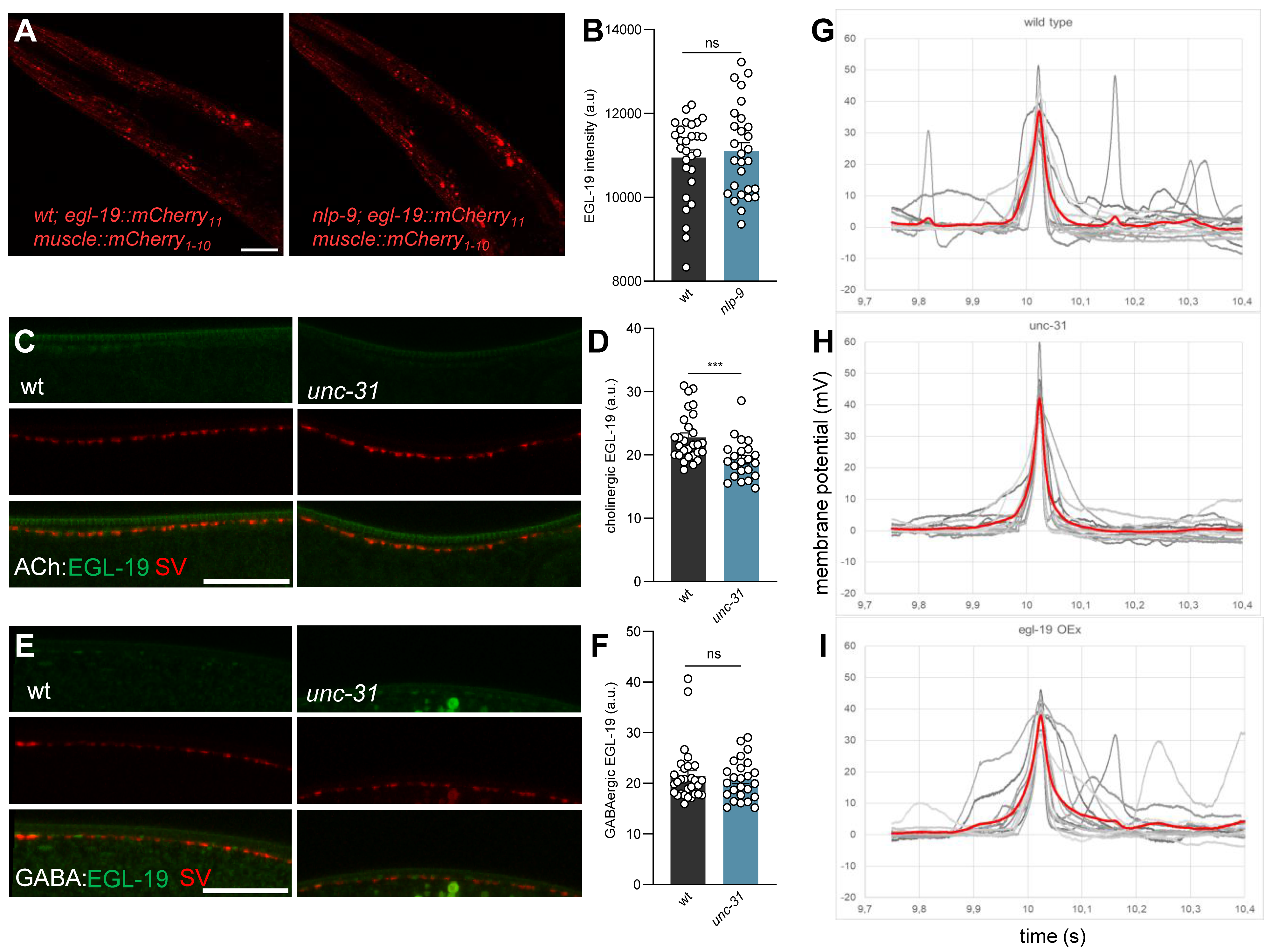

Supplement: S9 Fig — (A, B) Endogenous expression levels of EGL-19 in muscle cells were compared in wt (n = 14) and nlp-9 mutants (n = 14). Representative images (A; Scale bar 20 µm) and summary data (B) are shown. (C–F) Expression of endogenously GFP-tagged EGL-19 in cholinergic (C, D) and GABAergic neurons (E, F) were quantified in wt (n = 28 and 22, respectively) and unc-31 mutants (n = 29 and 25, respectively). Representative images (C, E; Scale bar 20 µm) and summary data (D, F) are shown. (G–I) Enhanced AP amplitude in unc-31 mutants; delayed and prolonged APs in animals overexpressing EGL-19 CaV1. Individual (gray) and mean (red) traces of membrane potential changes recorded from BWM cells after 20 pA current-step induced at 10.01 s, in wt (G), unc-31 (H), and EGL-19 over-expressing (OEx) animals (I) are shown, from n = 15, 15, and 14 animals, respectively. Note that the traces were aligned to the time of the peak amplitude, and replotted, thus they appear shifted to earlier times. All data are presented as mean ± SEM. Statistically significant comparisons were determined using unpaired t test. *** indicates p < 0.001. Numerical data can be found in S1 Dataset. (TIF) [file pbio.3003171.s009.tif]
