# Supplementary material for: Loss of neuropeptidergic regulation of cholinergic transmission induces homeostatic compensation in muscle cells to preserve synaptic strength
Source: PLoS Biol. 2025 May 8;23(5):e3003171. doi: 10.1371/journal.pbio.3003171 (PMC12088594; doi:10.1371/journal.pbio.3003171)
Supplement: S1 Table — (DOCX) [file pbio.3003171.s010.docx]

| neuropeptide | protein sequence | EGL-3 (PC2) cleavage sites | AEX-5 (PC1) cleavage sites |
| --- | --- | --- | --- |
|  |  | RR or KR | RXXR |
| *nlp-9a* | MDRFATRFIALLLVLLQIGSIFATPIAEAQGAPEDVDDRRELEKRGGARAFYGFYNAGNSKRDQAAALPYYLYEKRGGGRAFNHNANLFRFDKRGGGRAFAGSWSPYLERFYDYKRSSYPVYFSDNSYY | yes | no |
| *nlp-9b* | MDRFATRFIALLLVLLQIGSIFATPIAEAQGAPEDVDDRRELEKRGGARAFYGFYNAGNSKRDQAAALPYYLYEKRGGGRAFNHNANLFRFDKRGGGRAFAGSWSPYLERDNSYY | yes | no |
| *nlp-38a* | MQLIHFIVGLAMLISLSLAASDDRVLGWNKAHGLWGKRSVQEASQDKRTPQNWNKLNSLWGKRSASSFDDDYTTENGDDDVTMLYKRSPAQWQRANGLWGR | yes | no |
| *nlp-38b* | MQLIHFIVGLAMLISLSLAASDDRVLGWNKAHGLWGKRSVQEASQDKRTPQNWNKLNSLWGKRSASSFDDDYTTENGDDDVTMLYKRSNLSPRFLGRMTFARIPKISPAQWQRANGLWGR | yes | no |
| *flp-15* | MQFSTLIRVAVFAVLAIATLADYDDNSVGTIPVAVDLDYFSNYVKKGGPQGPLRFGKRRGPSGPLRFGKRSSFHVAPAAEDVASWYQ | yes | no |
| *nlp-15* | MPSSSSSSSFFAAVLLVIVMMSTVESAAVRLRPVGSLFFLNRPHEKRAFDSLAGSGFDNGFNKRAFDSLAGSGFGAFNKRAFDSLAGSGFGAFNKRAFDSLAGSGFSGFDKRAFDSLAGQGFTGFEKRAFDTVSTSGFDDFKL | yes | no |
| *nlp-21a* | MRNSLFTTLFFGLAALVMVLNAQYTSELEEDEKRGGARAMLHKRGGARAFSADVGDDYKRGGARAFYDEKRGGARAFLTEMKRGGARVFQGFEDEKRGGARAFMMDKRGGGRAFGDMMKRGGARAFVENSKRDEDWVIRPFEDDRLEKRGGGRSFPVKPGRLDD | yes | no |
| *nlp-21b* | MRNSLFTTLFFGLAALVMVLNAQYTSELEEDEKRGGARAMLHKRGGARAFSADVGDDYKRGGARAFYDEKRGGARAFLTEMKRGGARVFQGFEDEKRGGARAFMMDKRGGGRAFGDMMKRGGARAFVENSKRDEDWVIRPFEDDRLGVF | yes | no |
| *nlp-12* | MLRHHSCALLMLILVFVEVFATQSPTFDRQDRDYRPLQFGKRDGYRPLQFGKRDYRPLQFGKRSSGSSGPVVLEPIWEWQ | yes | yes |
