# Supplementary material for: Loss of neuropeptidergic regulation of cholinergic transmission induces homeostatic compensation in muscle cells to preserve synaptic strength
Source: PLoS Biol. 2025 May 8;23(5):e3003171. doi: 10.1371/journal.pbio.3003171 (PMC12088594; doi:10.1371/journal.pbio.3003171)
Supplement: S3 Table — (DOCX) [file pbio.3003171.s012.docx]

Table S3

| Primer | Sequence (5’→3’) | Description |
| --- | --- | --- |
| oJS92 | ccggctagcGGGTCTACAAAACAAAAG | for amplification of *nlp-38* gDNA from N2 genome |
| oJS93 | cggggtaccCACGAAAAACATATTCATTT |  |
| oJS95 | tgattacgccaagcttgcatgctTGCCGAAGTTTTAAGTTAA | for amplification of *nlp-38* promoter from N2 genome |
| oJS96 | aatcccttgaacaagagatgcggTGGAAAAAAATGGAATTTGG |  |
| oJS113 | tttcaggaggacccttggctagcAGAAGAAAAAAGAAAAAAAGAGAGATG | for amplification of *nlp-9* gDNA from N2 genome |
| oJS114 | aagtaggatgagacagcggtaccATTCACATAGCAAATTTTATTGCAA |  |
| oJS118 | acatgcatgcTGCAGAGAAGAGGTAGGTAGGTTT | for amplification of *nlp-9* promoter from N2 genome |
| oJS119 | tcactggccaCTGAAAATTTGAAGTTCAAGGCGTATGA |  |
| oJS162 | cccaagcttGGATCATTCTGTCGAAATGCATTC | for amplification of *egl-19* RNAi fragment from N2 genome |
| oJS163 | ccggctagcAGGGAACGAATGGAAGTTATTGTT |  |
| oJS193 | ccggctagcaaaaATGTCGAATG | for amplification of *unc-31* cDNA from KG#121 |
| oJS194 | cggggtaccatTTAATGTTTTCGTATACCTTCTTG |  |
| oJS246 | acatgcatgcGAAGACCGACGACTTGG | for amplification of *aex-5* promoter from N2 genome |
| oJS247 | tcactggccaTAAAAATTGCGGTCATAAACTG |  |
| oJS233 | ccggctagcATGAAATTAATTTTCCTGCTTTTG | for amplification of *aex-5* cDNA from N2 genome |
| oJS234 | cggggtaccTTATGACATTGTTCCCACCA |  |
| oJS321 | cactagatccatctagTCTAGAGATGTCAGTGTTAGCGAGTA | for amplification of *egl-19b* (two fragments) from KP#2460 |
| oJS322 | caggctgaagtttgtggcggtaccAAGAGTTGTAACTAAAAGTAGATC |  |
| oJS325 | ATGTTAATGATACTGCAACGA |  |
| oJS326 | CGTCGTTGCAGTATCATTAA |  |
| oJS354 | cgtcacatttatttcattacagtATGAAAAACACACATGTCGACCT | for amplification of *egl-3 gDNA* from pJH2124 |
| oJS355 | caggctgaagtttgtggcggtaccGTGGCTGCGTTTGTGGGC |  |
