# Supplementary material for: Loss of neuropeptidergic regulation of cholinergic transmission induces homeostatic compensation in muscle cells to preserve synaptic strength
Source: PLoS Biol. 2025 May 8;23(5):e3003171. doi: 10.1371/journal.pbio.3003171 (PMC12088594; doi:10.1371/journal.pbio.3003171)
Supplement: Attachment — Submitted filename: responses to the reviewers.pdf [file pbio.3003171.s026.pdf]

Dear reviewers,

thank you for your latest perusal of our manuscript! We have addressed *your points* (explained below), and now hope that we could resolve all remaining issues.

On behalf of the authors,

Alexander Gottschalk

Reviewer remarks:

Reviewer #1 (Shangbang Gao): This paper represents a significant contribution to the field, offering valuable insights for assessing compensatory neural functional changes. The authors have adequately addressed my concerns and has supplemented the necessary experiments or provided additional explanations. I have no further questions.

Thank you again!

Reviewer #2: A couple of points from the first review were not adequately addressed.

Relevant to previous Major Comment 1: The rationale for varying stimulation frequency was explained in the rebuttal, but we could not find it in the manuscript itself. The manuscript would benefit from a clear explanation for how and why stimulation frequencies were chosen for the various genotypes used. Summary data were not labeled as being an average over the entire stimulation time in the figures or figure legends in the revised manuscript, even though the rebuttal states that it was. This should be added to the figure legends.

Apologies, we now discussed the reasoning for using different intensities at three places in the manuscript. Also, we completed the information regarding the time windows averaged in each figure legend. We had done this in some and overlooked it in others.

Relevant to previous Major Comment 3: Figure 6 now more clearly labels that the methods used in Figure 6A are bPAC expression in cholinergic neurons. However, the X-axes in Figure 6H and I confusingly label different aspects of the experiment (light stimulation and genotypes). Both panels should have both sets of labels as there is space available to do so.

Thank you for spotting this, we adjusted this now.

Additional comment: In Figure 4, relative body length recordings and respective quantifications do not match. Figure 4D belongs next to Figure 4G, Figure 4H belongs next to Figure 4I, and Figure 4J belongs next to Figure 4C. This likely oversight should be corrected to avoid confusion.

Oh, yes, indeed, this was misplaced. Thank you, we adjusted this.

Reviewer #3: The authors have addressed most of my prior comments. I have only a few suggestions for additional clarification.

Lines 78-79: I would suggest the authors amend this sentence to state, " A major mode of action of these peptides…." or similar, as it is unlikely that modulation of SV filling is their exclusive mode of action.

Thank you we added your suggestion.

Line 96: missing a "we"

Line 244: I think the authors are referring to 2C here instead of 1C.

Thank you for spotting these two points, we adjusted them.
