# Supplementary material for: Loss of neuropeptidergic regulation of cholinergic transmission induces homeostatic compensation in muscle cells to preserve synaptic strength
Source: PLoS Biol. 2025 May 8;23(5):e3003171. doi: 10.1371/journal.pbio.3003171 (PMC12088594; doi:10.1371/journal.pbio.3003171)
Supplement: Attachment — Submitted filename: Rebuttal letter PLoS Biology final 28 2 25.docx [file pbio.3003171.s025.docx]

**Rebuttal letter PLoS Biology 26.2.2025**

Dear Dr. Schnell,

we would like to thank the reviewers and you for your thoughtful advice and suggestions, and for your expert handling of our manuscript review. We addressed most of the requests, which allowed us to substantially improve the manuscript. We will describe the new experiments below, answering to each point of the reviewers.

**Reviewer #1 (Shangbang Gao):** This manuscript offers an innovative exploration of how neuropeptidergic regulation impacts cholinergic transmission and the resulting compensatory mechanisms in muscle cells in C. elegans. The authors present a novel finding that reduced acetylcholine output due to impaired presynaptic neuropeptide signaling triggers homeostatic upregulation of the L-type voltage-gated calcium channel EGL-19 in postsynaptic muscle cells, effectively preserving synaptic strength. This discovery adds a new layer to our understanding of synaptic plasticity and highlights the intricate balance between pre- and post-synaptic mechanisms in maintaining neuromuscular function. Overall, this study provides significant insights and a valuable contribution to the field of neurobiology.

Thank you, Prof. Gao, for your positive feedback!

Major Comments:

1. The proposed mechanism of postsynaptic homeostatic scaling, mediated by increased muscle excitability via upregulation of L-VGCC/EGL-19, is a key and intriguing finding. This compensatory mechanism response to reduced ACh-output in neuropeptide signaling mutants is central to this study. However, an important question arises: is this a general principle across different contexts of reduced cholinergic transmission? For instance, would similar postsynaptic compensation occur in other mutants with impaired cholinergic transmission? I was curious to ask if this phenomenon is also present in mutants such as unc-13, unc-17, or unc-2 etc. ? Additional experiments addressing this question would greatly enhance our understanding of whether the observed EGL-19 upregulation is solely due to reduced ACh-output or if other contributing factors co-exist.

We have provided an experiment addressing this in the first version of the paper already, i.e. in showing that upregulating the ACh levels by mutating acetylcholine esterases could recover the enhanced excitability of muscles in the *unc-31* background (formerly Fig. 6L, M). Also, we have done such experiments in 2008 (Liewald et al., 2008, *Nat Meth* **5:** 895-902, Fig. S5) i.e. showing that in *unc-13* and in *snb-1* mutants with reduced neurotransmitter release (including ACh release at the NMJ), less optogenetic depolarization is required to achieve the same muscle contraction. This paper was cited in the present manuscript. We now added another experiment to this in reducing ACh levels in a r.o.f mutant of choline-acetyltransferase, CHA-1. This manipulation likewise increased muscle excitability, to the same extent as *unc-31* mutants (this is now in Fig. 7E, F).

2. The authors mention that egl-19 is expressed in both presynaptic neurons and postsynaptic muscle cells. My second question: Is egl-19 also upregulated in presynaptic neurons in the unc-31 mutants and other neuropeptide mutants identified in this study? While this question may seem outside the primary scope of this study, addressing this is crucial for a complete understanding of the proposed conclusions. If egl-19 is similarly altered in presynaptic neurons, it could indicate that neuropeptides directly regulate egl-19 expression in multiple tissues. This would help clarify that the behavioral effects observed in the mutants are not solely due to increased egl-19 expression in muscle cells. Surely, it is possible that neuropeptides use distinct mechanisms to regulate egl-19 in nerve and muscle cells. If so, wouldn't that further expand the depth of research in this paper!

We did the suggested experiments. EGL-19 is not upregulated in neurons, in the *unc-31* mutant, but either unaffected (in GABAergic neurons), or even downregulated (in cholinergic neurons). This may indicate a reciprocal regulation in the excitatory branch of overall E/I balance. In the *nlp-9* mutant, we did not see any effect on EGL-19 levels in muscle. Likely, this is because it requires the absence of more than just one neuropeptide (like in the *unc-31* background), or, it is another of the peptides we studied in the present version of the paper, that all contributed to regulating muscle excitability and/or ACh release (NLP-9, NLP-21, NLP-38, NLP-15, FLP-15). These new experiments are in Fig. S9.

3. The use of the Ach::bPAC optogenetic method to demonstrate reduced mPSCs in nlp-9 and nlp-38 mutants is compelling. However, several points warrant clarification: Do unc-31, egl-3, and axe-5 mutants exhibit similar declines in mPSCs? What was the rationale behind using the Ach::bPAC optogenetic method for this experiment? Are endogenous mPSCs and EPSCs (evoked via ACh::ChR2 stimulation) similarly reduced in mutants such as egl-3, axe-5, nlp-9, nlp-38, FLP-15, and NLP-15? These clarifications would be critical to validate the reduction in ACh-output in the mutants analyzed.

We chose ACh::bPAC as it is not possible to induce neuropeptide release from cholinergic neurons otherwise (ChR2 stimulation), as we showed in our publication in 2017 (Steuer Costa et al., 2017, *Curr Biol*, **27**: 495-507): depolarization alone was not sufficient, it required cAMP increase. We assessed *aex-5; egl-3* double mutants, and found that they similarly affected presynaptic ACh release, just like *unc-31* mutants (in Fig. 6B, C, H, I). We assessed ChR2-evoked EPSCs in *unc-31* mutants (which were reduced; Fig. S1C, D), while mPSCs were not affected (Fig. S7A). We tested bPAC-induced effects in flp-15; nlp-15 double mutants, which were compromised, despite an intriguing larger basal mPSC amplitude in these mutants, indicating that these neuropeptides expressed in GABAergic neurons affect inhibition. These data are in Fig. 6A, F, G-I, as well as in S6. We did not perform all the ChR2 experiments requested, as these could not be done within the given timeframe for revision. Also given the conclusiveness of the extended data we provide, it is not conceivable that new information would be gained from them.

4. The draft provided by the author lacks all supplementary figures, some of the data cannot be judged substantively.

This is not correct; the supplements were uploaded along with the main manuscript. We notified the editorial staff to convey this information, however, we could not do so before we received this review.

Minor Concerns:

1. It would be beneficial to examine whether the phenomenon of postsynaptic compensation observed in unc-31(n1304) can be consistently reproduced in different unc-31 mutant alleles. This would help to solidify the findings.

We tested a second allele, *e928*. The results are identical to *n1304* in ACh::bPAC-induced speed increase and muscular ChR2-induced body contraction (Fig. 1A, B, G, H; 3A-D).

2. In Fig. 2, egl-3 mutants exhibit enhanced muscle excitability even with milder stimulation. Could the authors elaborate on the potential mechanism driving this phenomenon? Additionally, a more detailed description of the types of neurons in which egl-3 primarily functions is needed .

This is probably simply a matter of finding the optimum in stimulus intensity, to uncover subtle differences among mutants. *egl-3* mutants showed enhanced muscle contraction when an intense stimulus (~ 0.1 mW/mm^2^) was applied (relative body length, wt 0.95 versus 0.94 for *egl-3*), although not significantly different from wt (Fig. 2C, D). We reasoned that EGL-3 convertases only process partial neuropeptides that are responsible for inducing postsynaptic compensation, or other convertases, e.g. AEX-5 could compensate for its loss. Thus either mutant showed only partially enhanced muscle contraction compared to *unc-31*. Subtle effects might be overridden when muscles are intensively activated. We therefore applied a milder stimulation (~ 0.065 mW/mm^2^) to *egl-3* mutants, which induced a significantly stronger muscle contraction compared to wt (relative body length, wt 0.96 versus 0.94 for *egl-3*, Fig. 2G, H). Further, we show now that EGL-3 act in both, cholinergic as well as GABAergic neurons, with a stronger contribution from cholinergic neurons, and suggesting that the rescuing effects are likely additive.

3. In Fig. 5B,C, Ach::bPAC activation results in a modest increase in NLP-9 secretion. Could the authors provide further explanation as to why this increase is weak? Is it possibly related to the duration of light stimulation ?

We are not sure what a modest increase is, in this context, and we do not know what a ‘strong’ increase would be, or the upper limit of the coelomocyte signal. As NLP-9 is likely released all the time during the life of the animal, upon normal locomotion, there will always be a certain base-line level of fluorescence in the coelomocytes. All we wanted to explore is whether blue light / bPAC can acutely induce additional release, that should result in increased coelomocyte signal, which it did. We are aware that, possibly, prolonged illumination, or imaging at a later time point may lead to different results, maybe a stronger increase, as we observed earlier for a similar experiment using NLP-21 as the reporter (Steuer-Costa et al., 2017, *Curr Biol*, **27**: 495-507). But this will depend strongly on the baseline level of release of the respective neuropeptide, and may be individually different.

4. In Fig. 5DE, does the nlp-9; nlp-38 double mutant show a further reduction in mPSCs? Additionally, does unc-31(lf) also reduce Ach::bPAC-excited mPSCs ?

We tested *nlp-9; nlp-21; nlp-38* triple mutants as NLP-21 is a very abundant neuropeptide in cholinergic neurons. The triple mutant is somewhat more affected, though there was no significant difference to the single mutants (Fig. 6D, E, H, I). Thus, possibly the effects are not additive, as we also observed in the behavioral levels (Fig. 3E and Fig. 4C, D).

5. In Fig. 8A, do other neuropeptide mutants exhibit upregulated endogenous levels of muscular EGL-19, similar to what is observed in unc-31(lf)? Investigating this would help establish whether this compensatory mechanism is generalizable across other mutants.

As we described above, we tested *nlp-9* mutants, which did not affect EGL-9 levels. Likely, as it requires multiple neuropeptides to be removed for these compensatory effects to become fully observable. At the behavioral level, *nlp-9* mutants showed enhanced muscular ChR2-induced body contraction only when the milder stimulation (0.065 mW/mm2) was applied (Fig. 4C, D), but not at the intense stimulation (0.1 mW/mm2, Fig. S4A, B). Likely, if there is a subtle change in muscular EGL-19 in *nlp-9* mutants, we may not be able to detect it using the endogenous fluorescence label.

6. In Fig. 8E, the action potentials do not fully resemble the standard waveforms previously recorded in similar studies. This discrepancy may have affected the analysis and contributed to inconsistencies in the text when comparing unc-31(lf) and EGL-19 OEx effects. It may be more informative to directly record voltage-gated calcium currents in WT, unc-31(lf), and EGL-19 OEx strains to assess whether functional calcium currents are indeed increased.

We appreciate the comment, but we are not experienced with methods for measuring Ca^2+^ currents. The mean AP waveforms in response to electrical stimulation, as used in other studies, result from the peak alignment that we performed. The individual APs are shown in Fig. S9 G-I. Importantly, we analyzed all genotypes side by side, thus the comparison of the data should be consistent.

Overall, this study presents several innovative and important findings on the role of neuropeptidergic regulation in synaptic plasticity. Addressing these points would further strengthen the conclusions and provide deeper insight into the mechanisms underlying cholinergic transmission and compensatory regulation in C. elegans muscle cells.

Thank you. We hope the added experiments do strengthen the conclusions also in the reviewers’ eyes.

**Reviewer #2:** In this manuscript, Shao et al. seek to determine whether neuropeptidergic signaling modulates synaptic transmission through homeostatic plasticity, specifically via the upregulation of postsynaptic CaV1 channels. They use optogenetic bPAC stimulation in cholinergic motor neurons to induce cAMP-dependent neuropeptide release, coupled with behavioral experiments highlighting the mutant phenotype of unc-31 (CAPS ortholog, responsible for dense core vesicle release). Rescue experiments indicate that a complex neuromodulatory interface exists between cholinergic and GABAergic neuropeptides at the neuromuscular junction (NMJ). The identification of AEX-5 and EGL-3 as key proprotein convertases that work jointly in UNC-31-dependent signaling at the NMJ is a noteworthy contribution. The study provides evidence that cholinergically expressed neuropeptides NLP-9 and NLP-38 modulate locomotion and muscle contraction. In addition, FLP-15 and NLP-15, highly expressed in

GABAergic neurons, also function to modulate muscle contractions. Overall, they find a compensatory enhancement in postsynaptic Ca2+ influx following the loss of UNC-31 using optogenetics, calcium imaging, and voltage imaging. They identify EGL-19, an L-type VGCC, as a critical player in this muscle homeostasis. While some experiments are hare to interpret, and there is a high degree of variability in many of the assays, overall, the findings are novel, and the following adjustments will make the contributions clearer to readers.

Major comments

1. Overall the high degree of variability in the phenotypes of even control strains from experiment to experiment makes the data a bit hard to interpret. For example, wt body length upon muscle ChR2 stimulation is around 0.95 in figure 1H, but closer to 0.98 in Fig. S3, where triple mutants are now at 0.95 (and concluded to be defective in their excitability). If the experimental variability between experimental sessions is as great as the variability between wt and mutant, how can the data be properly interpreted? A bit more discussion of what might be leading to this variability is necessary.

We now describe the methodology more detailed and clarify why there is data variability. This is mostly due to different intensities of light stimulation. We first analyzed the *unc-31(n1304)* mutants by muscular ChR2 stimulation with saturating light intensity (~ 1.4 mW/mm^2^: relative body length, wt ~ 0.9, formerly in Fig. 7A-D, now in Fig. 8A-D), or a medium light stimulus (~ 0.1 mW/mm^2^: relative body length, wt ~ 0.95, Fig 1. G-J). However, the single *nlp-9* mutants show similar relative body length at this intensity as compared to wt, Fig. S4A, B. Therefore, we applied a even lower stimulation at 0.065 mW/mm^2^, since we could observe a significant enhanced muscle contraction in *snb-1* mutant with such intensity in our previous report (Liewald et al., 2008, *Nat Meth* **5:** 895-902, Fig. S5). *nlp-9* mutants show an enhanced muscle contraction compared to wt with this low stimulation (wt 0.965 relative body length versus 0.93 for *nlp-9,* Fig. 4C, D). We applied this stimulation condition to all the other mutant analysis, as this brought out the differences better. Of course, we treated controls and test genotypes identical in each comparison / data set for a given figure. However, we now redid some experiments to include positive controls. i.e. *unc-31* in all cases, where appropriate.

In addition, how summary data were calculated is not clear (i.e.- how figure 1H was generated from figure 1G… is that an average over the entire time? It is the value at a particular time point?).

The average is over the entire stimulation time, i.e. sec 5-15. Apologies, we now labeled this in all figures or stated it in the figure legends.

2. Absence of unc-31 mutant controls in Figures 3D-E and 4C-H limits the interpretability of the data regarding neuropeptidergic modulation of muscle cell excitability. It would be informative to know how these individual peptide phenotypes compare to the unc-31 mutant phenotype. Moreover, the double (nlp-9; nlp-38) or triple mutants (nlp-9; nlp-21; nlp-38) mutant data should be presented alongside single mutant data (rather than separately in supplemental material) so they can be directly compared.

We re-measured the strains in Fig. 3D-E (now in Fig. 4I-J) and Fig. 4C-H together with *unc-31* mutant controls. Data for the double and triple mutants, originally in Fig. S3A, B, are now presented alongside single mutants in Fig. 4C, D.

3. The methods for the experiments shown in Figures 5D-H are not adequately described, specifically what is being expressed and where (ChR2? bPAC?) and what time points are the summary data in G-H being drawn from?

The cholinergic bPAC strain was used in Fig. 5D-H (now in Fig. 6). The original figures were modified for clarification. Summary data bars labeled with light – indicate the mean value of 1 - 30 s before blue light stimulation, and bars labeled with light + indicate the mean value of 31 – 55 s after stimulation. We now labeled this in the figure legend.

From panels E-F, it seems like the mutants are still responding initially to the stimulation, albeit more weakly, but that the response is not as sustained as in wt. However, the summary data suggest that the mutants have no change at all in mPSC amplitude or frequency upon stimulation. This is confusing.

Thanks for pointing out this. Due to the comparably high variability of the data, especially for mPSC rate, the initial, small response as apparent from the mean curve, does not come through as a statistically significant difference. We tested different time windows for the analysis, which did not make a difference, though. For the mPSC amplitudes, the data was clearer, and the differences of the mutants (smaller induced amplitudes) compared to wt were statistically significant (Fig. 6I). The non-normalized data is shown in Fig. S6.

4. The minimal effect observed in wild-type animals with both nemadipine and especially RNAi treatments is unexpected, given the central role of EGL-19 in muscle excitability. This suggests that only the "compensatory" channels are affected, a point that, if believed true, should be elaborated on in the discussion. The timing of the RNAi application is not clearly stated- is it added late in development or are the worms grown up on the RNAi plates? Could this play a role in the lack of effect in wt? Also, the specificity of the RNAi to the muscle EGL-19 (due to the lack of neuronal sensitivity to RNAi) should be mentioned.

We had cited the respective paper with the protocol (Wabnig et al., 2015), but we describe this now in detail in the methods section. We think that the RNAi knockdown cannot be complete, as those animals would be dead. This likely explains the small effects. For nemadipine, we are aware that stronger effects have been reported in the literature, but these were during electrophysiological recordings, in dissected animals, where there is free access of the drug to the muscle cell surface. We do not know if one can increase the amount of nemadipine to such a level that similar concentrations are achieved in the animal, or, we would have to add so much of the stock that a considerable concentration of DMSO would be reached, which is in itself deleterious to worms (above 1%, in or hands). We added a sentences in the discussion now. The idea of ‘compensatory’ channels is interesting, but we are not convinced that the manipulations of RNAi or nemadipine could distinguish these from other EGL-19 channels, or preferentially affect only these ‘compensatory’ channels.

Minor comments

1. The unc-31 mutant phenotype involves an emergence of a latent secondary peak in speed approximately 100 seconds after photostimulation, accompanied by an extended decay. The authors may consider elaborating on this phenomenon in the text to provide further insight.

We do not know why this delayed locomotion speed increase happens. We assume it to be a late effect of cAMP signaling / PKA activity, leading to a 2^nd^ sustained speed increase. We extracted more parameters from the multi worm tracker (MWT) data. Two of those parameters appeared to correlate with the 2^nd^ locomotion speed peak: 1) The ‘*angular speed’* (of objects tracked, in radians/sec; calculated over the same interval as speed, but reporting the greatest difference in angle between primary axes over that time). 2) The parameter *‘crab’* (the speed perpendicular to the length of the body, in mm/sec; the speed along the length of the body is sqrt (speed^2^ – crab^2^)*,* referring to relative sideways deflection of the animal trajectory.

Since the MWT does not save a video, we cannot see the animals. E.g., these two parameters could indicate an overall locomotion strategy change e.g. local search behavior as opposed to long range straight movement. Importantly, animals do not alter their speed if there is no bPAC photo-stimulation:

2. The study aimed to identify neuropeptides responsible for modulating cholinergic signaling by extracting expression pattern information from previously published data. However, the rationale for the selection of candidate neuropeptides from the CenGen scRNAseq database remains unclear.

We did not exactly understand this comment. The rationale is that we focused on those peptides that are abundantly expressed in cholinergic cells, as opposed to other cell types. In addition, as the circuit of the motorneurons includes GABAergic neurons, we also included such neuropeptides expressed in these cells. So we chose the neuropeptides most abundantly expressed in either cholinergic or GABAergic neurons. This should become obvious from the supplementary Fig. S3.

Also NLP-12 is said to have previously been studied, but neither context nor citation is included.

We included data for NLP-12, and referenced the publication that first analyzed NLP-12 (Hu et al., 2011). This was already present in the first version of the paper, just not in line 327 (lines 77, 98, etc.).

3. A distinction should be made between when something is "significantly" increased or decreased (which means there is a large difference) versus when there are small but "statistically significant" differences. At several points in the text this distinction is lost, for example, lines 378 and 540, where a small (albeit statistically significant) difference is described as being "significant" (ie- large).

Yes, apologies, of course we are aware of this distinction. We now clarified this throughout.

4. In figure 5E-H, the Y-axis is labeled "mPSC," even though blue light is being used to evoke a response, the response is clearly larger than individual quantal events, and the text on line 383 refers to them as "evoked" postsynaptic currents. A clearer distinction between (or definition of) mPSC and evoked PSC is needed in both the figures and the manuscript text.

We tried to be clearer about this, and added an explanation. Some of the increase may be due to (unresolved) multivesicular fusion events; we still refer to these as mPSCs, because the underlying SV fusions are not evoked by bPAC stimulation, rather, the induced cAMP signaling affects the rate of priming and mobilization of SVs. Thus intrinsic activity of the neurons leads to more SV fusions per time. By this definition, evoked PSCs are due to SV fusion events induced by depolarization of the neuron, or by applying ACh to trigger AChRs.

5. Inconsistency in stimulation duration (ranging from 10 to 40 seconds) across optogenetics assays needs justification. Standardizing this protocol would enhance the comparability of results.

For ACh::bPAC assays, we used 5 s stimulation in the locomotion speed assay, and 40 s for contraction assay, as this provided more robust difference between wt and mutants. For ACh::ChR2 assays, we also used 5 s stimulation to minimize the worm coiling during cholinergic neuron activation, which otherwise precludes automated analysis. For muscular ChR2 contraction assay, we initially applied a 30 s (5 – 35 s) stimulation (formerly in Fig. 7A-D, now in Fig. 8A-D). Later we shortened the stimulation time to 10 s (5 – 15 s) as worms already reached the maximum muscle contraction after 1 s stimulation, to save experimental time. Nevertheless, we partly repeated some of the experiments analyzed the body contraction of 5 – 15 s in Fig. 8A-D as in other Figures, which shows similar results as of 5 – 35 s.

Rebuttal figure: analysis of relative body length of 5 – 15 s from Fig. 8A, C.

6. The novelty and impact of the manuscript would shine with revisions for clarity and succinctness by simplifying complex sentences and removing any redundancy.

We tried to do this as much as possible.

7. The final figure summarizing the proposed model would greatly benefit from a more descriptive title and perhaps a simpler layout. For example, removing the DVA neuron, which was not discussed in detail , and using a distinct symbol for autocrine release would improve clarity. A subcellular view of the model , in addition to the current circuit view, might better capture the detailed mechanisms explored in the manuscript.

We modified the figure and provided a descriptive title. We prefer to retain the DVA neuron, as the optogenetic manipulations we do will also affect DVA, and as it has been shown to have clear functional influence on motor neurons, based on neuropeptide signaling (Hu et al., 2011). Besides, it is included in our candidate screen. We also discuss DVA and its role in the paper, so it should simply not be overlooked when regarding NMJ modulation by neuropeptides. We chose a distinct symbol now autocrine and paracrine signaling.

As for the subcellular aspects, essentially our work is on the circuit level, and we do not know too much about the cell biological details / signaling that leads to e.g. increase expression of EGL-19. Thus we kept this simple. We prepared a second panel for these aspects (see below), but as this contains a lot of unknowns, we need to keep a lot of question marks emphasizing these open points, and thus would prefer not to include this in the final version of the paper.

**Reviewer #3:** This is an interesting study from Gottschalk and colleagues that aims to dissect the interplay of pre- versus postsynaptic impacts of neuropeptide (NP) signaling at the C. elegans NMJ. The work builds from a prior 2017 publication from the same lab that provides evidence for cholinergic presynaptic modulation through neuropeptide regulation of vesicular loading. The current study extends these findings to explore locomotory effects of NP modulation, identify specific neuropeptides involved, and define a potential pathway for postsynaptic homeostatic compensation through upregulation of EGL-19 L-type voltage-gated calcium channels in the muscles of NP-deficient mutants. The overall idea is intriguing and the experimental results presented largely support the authors' model; however, several of the assays are indirect or employ different methodologies across experiments, in some cases making it difficult to draw strong conclusions. As just one example, the authors often switch between presynaptic bPAC (cAMP) stimulation for the behavioral studies and ChR2 stimulation for the electrophysiology studies , making it very difficult to relate the 2 sets of findings. There are a number of interesting results here, but alternative models that are not deeply explored could also account for many of the findings . I provide detailed comments below.

1. Figures 1A,E: It would be interesting to show whether ChR2 stimulation of cholinergic motor neurons elicits the same increases. If the effects are mediated through postsynaptic compensation due to loss of NP signaling, I would predict a similar behavioral response and increase in muscle calcium for ChR2 stimulation of motor neurons in unc-31 mutants.

Thank you for bringing up that this was apparently unclear. We have looked at ChR2 stimulation in cholinergic neurons earlier, i.e. Liewald et al., 2008, Nat Meth **5:** 895-902. The reason why we did not assess effects on locomotion behavior following ChR2 stimulation is that the stimulus is way too strong. ChR2 activation evokes massive fusion of (probably all primed) SVs, and leads to hypercontraction of the animals, which strongly hampers locomotion. The animals basically go into spastic paralysis; see also Kittelmann 2013, *PNAS* **110**: E3007-3016. bPAC stimulation instead leads to enhanced SV fusion rates based on increased SV priming and mobilization from the reserve pool, while fusion still requires depolarization that is provided by intrinsic motor neuron activity. Thus, bPAC stimulation accentuates / intensifies neuronal output, yet maintaining the coordinated activity of the neurons, while ChR2 activation just overrides any intrinsic activity patterns. Therefore, bPAC activation allows analyzing changes in the increased locomotion speed, while ChR2 does not.

As for Ca^2+^ imaging in response to ChR2 stimulation, we had done so and this was included in Fig. 6C, D, now Fig. 7C, D.

We also now provide an experiment using ChR2 in *unc-31* mutants, while recording postsynaptic currents. This is shown in Fig. S1C, D. Evoked responses are much reduced in *unc-31* mutants, which, however, is a very different stimulation, i.e. causing acute fusion, and not the buildup of SV fusions following cAMP/PKA induced mobilization and priming.

2. For many of the experiments, the authors show rescue with ACh-specific unc-31 expression. While this shows sufficiency, experiments aimed at showing necessity in ACh motor neurons would strengthen the analysis considerably. Using a reagent such as the available unc-31::AID strain for this purpose would also provide temporal control for demonstrating post-developmental roles, for instance in the case of EGL-19 upregulation in unc-31 mutants (Figure 8).

We acknowledge that this is a very relevant comment. We tried to do this AID experiment for the EGL-19 expression, however the complexity of the transgenes / genetic modifications that required being crossed prevented this. We did not get the strain.

We depleted UNC-31 using the AID system, though, specifically in cholinergic neurons, in the context of testing enhanced muscle excitability induced by muscular ChR2 activation. This showed the same outcome as the systemic *unc-31* knockout. This data is provided in Fig. 1K, L. We also acutely applied auxin or ethanol to adult worms for 5 h. UNC-31 levels were statistically significantly reduced by auxin, but not by ethanol exposure (Fig. S1G, H). Yet, the muscular ChR2-induced contraction was not affected by acute (5 h) application of auxin (Fig. 1K, L), suggesting that muscle excitability was not altered after post-developmental depletion of UNC-31. Collectively, these results demonstrate that *unc-31* must be absent during development (or >5h) in cholinergic neurons to induce enhanced muscle contractions.

3. In figure 3, different sets of NP-deficient mutants are surveyed across the 2 behavioral assays. It would be more informative if all of the mutants were included for each assay, as this would provide evidence for specificity of the effects.

We now included all neuropeptide mutants in the bPAC behavioral assay in Fig. 3C-E, including also mutants that were previously not analyzed in this assay (e.g., *flp-15; nlp-15*, or *nlp-9; nlp-21; nlp-38*).

4. Different experimental paradigms are used across many of the figures, making comparisons challenging and weakening the strength of some of the conclusions. For example, including recordings of evoked current responses to ChR2 photostimulation of motor neurons in Figure 6 would allow for direct comparison with the ACh-evoked response in panels 6H-I, and enhance the impact of the lack of an effect on the ACh-evoked response.

We included now a current recording from ChR2-stimulated animals, in wt and *unc-31*, in Fig. S1C, D. Due to the descriptive flow of the manuscript, we show it at this point, not in Fig 6. Still, just from the amplitude oof the ChR2-evoked response compared to the increase in the rate of minis evoked b bPAC, it is very obvious that these are very different stimuli (see also discussion above).

Also, inclusion of a current clamp recording of muscle APs in response to ChR2 stimulation of motor neurons would strengthen the argument that a reduced current elicits increased levels of muscle excitation in unc-31 mutants.

Since we performed current clamp in response to electrical stimulation, we do not think using ChR2 would add to this. Compared to the electrode, ChR2 evoked stimulation is less precise as it may be affected by cell to cell differences in ChR2 expression in a single cell experiment (this is less of a concern when looking at behavior, which is collectively evoked in many muscle cells).

Finally, a voltage clamp recording from muscles showing increased calcium current density would be the most effective way to demonstrate upregulation of EGL-19. The increase in AP amplitude in Figure 8E could arise through a variety of mechanisms. I am not suggesting that that authors need to perform all of these experiments, but instead pointing out that that the indirect nature of some of the approaches used and the accompanying limitations in interpretation should be noted more explicitly.

Regarding the suggested Ca^2+^ current recording, we agree this would be very helpful. However as described above, we are not familiar with the technique and establishing all conditions and controls was not possible within the given timeline for revision, along with the many other electrophysiological recordings that had to be performed. The fact that we observe larger EGL-19 fluorescent signal, and larger AP area under the curve speaks for EGL-19 mediated Ca^2+^ currents, though the contribution of other voltage gated ion channels like potassium channels, if reduced, may also have this effect. We refer to this in the discussion (lines 662-678).

5. Since the authors have not defined cognate receptors and their expression, it is difficult to rule out the possibility that changes in muscle excitability could arise due to direct effects of NPs on muscle. In particular, the effects of NPs thought to be released from GABA motor neurons are confusing. It seems the authors are suggesting GABA neuron-released peptides are required to sustain ACh transmission by acting to promote the filling of cholinergic SVs. While interesting, additional work is required to better support this model.

We consider it unlikely that neuropeptides directly affect muscle excitability via their cognate receptors. Increasing the presynaptic ACh levels by mutating ACh esterases in *unc-31* mutants could fully reverted the enhanced muscle excitability to wt level (Fig. 7N, O), suggesting that neuropeptides, either cholinergic or GABAergic, act presynaptically on regulating ACh release, not postsynaptically.

Nevertheless, it would be informative to identify the cognate receptors and their sites of action. We studied mutants in the following neuropeptide receptors: DMSR-7 (responding to numerous FLP neuropeptides, including FLP-15), NPR-18 (NLP-9), SPRR-1 (NLP-42, as a negative control) and SPRR-2 (NLP-38). Their expression patterns can be deduced from scRNAseq data, and here, SPRR-1 and DMSR-7 were detected in muscular tissue. However, these are enteric and vulval muscles, i.e. muscles not relevant for locomotion. All receptors are expressed in multiple neurons, including one or more classes of motor neurons. Thus, also because of this we are confident that we can exclude a muscular site of action. As to the precise location of the receptors with relevance to the mutant phenotypes we observed for the respective peptides (cognate peptide-receptor pairing is sometimes ambiguous, i.e. multiple FLP peptides activate DMSR-7), clearly, much more work is required to understand the details.

6. Mechanisms that link autocrine effects of NPs with SV filling are left unexplored. Likewise, potential mechanisms for EGL-19 upregulation are not explored. These could easily be topics for future standalone papers, but are nonetheless areas where there is a lack of clarity in the present study. Collectively, this leaves the reader with the impression that the results are mostly considered in the context of a pre-existing framework with less attention to other possibilities .

We understand the suggestion to be more cautious with our interpretation. We think that the autocrine function became relatively clear from our preceding work (Steuer Costa, *Curr Biol*, 2017): SVs were loaded with more ACh in response to bPAC activation in the cholinergic neurons, this was abolished in *unc-31* mutants, as well as by a drug affecting the vesicular ACh transporter. Of course, in the absence of a molecular mechanism in neurons, leading to increased activity of the vAChT, we can’t be too sure. Such mechanisms are likely to involve G-protein pathways downstream of the neuropeptide receptors for the studied neuropeptides, provided they are expressed in the motor neurons that release these peptides. This is a major task for future work. The second unknown becoming apparent from our results is how muscle alters its expression of EGL-19 in response to the reduced amount of ACh contained in the presynaptic SVs. Again, this may be through GPCR signaling (maybe, metabotropic AChRs), and downstream signaling affecting gene expression. Yet, again these pathways are as yet unclear and will have to be tackled in future work. We toned down our conclusions; lines (first paragraph of, and throughout, the discussion).

Minor comments

1. The authors should provide evidence that bPAC and/or ChR2 expression is not impacted in the various mutants, at a minimum for unc-31.

We provide this quantitative data now, in Fig. S1A, B, E, F; S7B.

2. It would be more clear if rescue strains were denoted with (+). For example, in Figure 2E: aex-5;ACh::aex-5(+)

We have done this throughout now.

3. Line 283 and elsewhere: The authors could perhaps be a little more cautious in relating the CenGen scRNA-seq data to protein expression. While the CenGen data are undoubtedly an incredible resource, they are unlikely to completely capture native protein expression across cell types .

True, we reworded our mentions of CenGEN data and discussion accordingly. It would need antibodies.

4. Line 291: "GABAergic FLP-15" is confusing. If it is also in other neurons, it is not exclusively GABAergic. The authors should rephrase .

Yes, true, we wrote this more precisely.

5. Did the authors examine impacts of flp-15;nlp-15 double mutants since the single mutants each elicit partial effects compared to unc-31 (Figure 3D,E versus Fig 2A-D ). Also, it looks as if the same data for unc-31 may be used for comparison across panels 2A and 2C. If this is the case, it should be noted in the figure legend.

We now assessed *flp-15; nlp-15* double mutants by behavior (Fig. 3C-E) and electrophysiology (Fig. 7). We rearranged and partially re-acquired data, noting if control datasets were used more than once.

6. Line 335: this experiment measures muscle contraction which the authors interpret as a proxy for muscle excitability. Stating the measurement as "muscle contraction" would be more clear.

Yes, understood, we reworded this throughout. Though we think that given the findings for EGL-19 and the evoked APs, one can really relate this to muscle excitability. We understand muscle excitability as the properties of the muscle membrane with respect to ACh- and voltage-evoked currents, that subject to the combined expression levels and properties of AChRs, VGCCs, and voltage gated K^+^-channels. As we see an effect for EGL-19, we would argue, this is relating to the excitability of muscle. Of course, contraction is a proxy, and potentially, activity of the intracellular Ca2+ channel UNC-68 (ryanodine receptor), as well as the muscle contractile apparatus itself, could contribute to the altered muscle contractions we observed. It will be a subject for a future work to explore all these additional potential contributors.

7. Lines 355-356: cell type specific rescue shows sufficiency, not a requirement.

Thank you, we changed this.

8. Figure 5D-H: It is not stated whether these recordings are measuring responses to ChR2 or bPAC stimulation. I interpret the data as ChR2 stimulation. As stated above, it is difficult to relate the bPAC-elicited behavioral responses and the Chr2-elicited electrophysiology responses . In figure 5G, it is unclear what statistical comparison the single asterisk refers to.

Indeed, we are sorry this was overlooked. It is now clarified in the legend and manuscript text. Fig. 5G is now renewed and this statistical comparison has changed anyway.

9. The most appropriate citation for characterization of CKR-1 as an NLP-12 receptor is Ramachandran et al elife, 2021 as cited in the Beets 2023 paper.

We included this now, thank you for pointing this out.
